# Pleomorphism in Wild-Type and Engineered PP7 Virus-Like Particles

**DOI:** 10.1002/smll.202506285

**Published:** 2025-09-30

**Authors:** Parisa Keshavarz-Joud, Matthew C. Jenkins, Tahiti Dutta, Liangjun Zhao, Carolina Hernandez, Daija Bobe, Mohammadreza Paraan, M.G. Finn, Mykhailo Kopylov

**Affiliations:** School of Chemistry and Biochemistry, Georgia Institute of Technology, 901 Atlantic Drive, Atlanta, GA 30306, USA; School of Chemistry and Biochemistry, Georgia Institute of Technology, 901 Atlantic Drive, Atlanta, GA 30306, USA; School of Chemistry and Biochemistry, Georgia Institute of Technology, 901 Atlantic Drive, Atlanta, GA 30306, USA; School of Chemistry and Biochemistry, Georgia Institute of Technology, 901 Atlantic Drive, Atlanta, GA 30306, USA; New York Structural Biology Center, 89 Convent Ave, New York, NY 10027, USA; New York Structural Biology Center, 89 Convent Ave, New York, NY 10027, USA; New York Structural Biology Center, 89 Convent Ave, New York, NY 10027, USA; School of Chemistry and Biochemistry, School of Biological Sciences, Georgia Institute of Technology, 901 Atlantic Drive, Atlanta, GA 30306, USA; New York Structural Biology Center, 89 Convent Ave, New York, NY 10027, USA

**Keywords:** cryo electron microscopy, structural diversity, structure, virus-like particles

## Abstract

Virus-like particles (VLPs) find applications across many different fields, aided by their stability, capacity for large-scale production, and presumed structural homogeneity. These attributes are a result of their highly efficient self-assembly, which stems from the evolutionary pressures on the natural viruses from which they are derived. It is found that VLPs based on the *Leviphage* PP7 assemble in an unexpectedly wide range of morphologies. The relative abundance of these structures is sensitive to small changes in the coat protein sequence. These results raise the possibility that structural plasticity may be a general property of such self-assembling structures rather than an exception.

## 1. Introduction

Biology is replete with molecules that self-assemble into larger, more complex structures. Viral formation is a remarkable example, in which hundreds of copies of only one or a few structural protein subunits create highly ordered shells to protect the viral genome. Common characteristics of such assemblies include balanced affinities between protein subunits,^[1]^ electrostatic interactions between protein and genomic material,^[2–4]^ rapid formation of oligomeric structures that represent stable intermediates in self-assembly,^[5,6]^ and strong thermodynamic stabilization of the resulting closed structures^[7,8]^ that are usually spherical or rod-shaped.

Viruses have evolved highly efficient ways to package genomic information with minimum resources. Self-assembling viral capsid proteins often form pentameric and hexameric units that combine in different ratios to form closed cages, pre-dominantly with icosahedral symmetry.^[9]^ The simplest icosahedral structure (designated as *T* = 1 triangulation number in Caspar-Klug nomenclature^[10]^) uses only 12 pentamers assembled from 60 identical subunits; higher *T* icosahedral symmetries assemble from 60*T subunits made up of 12 pentamers and 10*(*T*-1) hexamers.^[10]^ Tubular structures are usually folded sheets of hexameric subunits with hemispheric 5-fold symmetric caps at their ends.^[11,12]^

Virus-like particles (VLPs) are protein nanocages derived from natural viruses, which undergo self-assembly similarly to natural virions but do not contain the genomic DNA or RNA needed for self-replication. The viral genome often plays a role in templating or regulating assembly,^[3,4,13–15]^ but random polynucleotide^[16]^ or even synthetic polyanions^[17]^ can serve a similar purpose. VLPs typically range from 20 to 200 nm in diameter and can be recombinantly expressed in high yields.^[18]^ They are stable under a wide range of temperature,^[19]^ pH, and solvent conditions,^[20]^ especially if they are derived from bacteriophages. Among their useful features is the ability to package different types of cargo^[21]^ and to be amenable to genetic,^[22,23]^ chemical,^[24,25]^ and enzymatic modification,^[26]^ making them suitable candidates for applications from drug delivery^[27,28]^ to immunology^[29–32]^ and imaging.^[33–35]^

While protein cages are often assumed to be structurally uniform, there is growing evidence that pleomorphism—the formation of multiple structures from the same building blocks—is quite common. For example, pleomorphism can play an important role in viral fitness as in the case of multipartite viruses that form cages of different sizes to encapsulate different components of the viral genome.^[36]^ Some VLPs are known to assemble into capsids with different symmetry states either spontaneously or in response to the demands of different cargo.^[37–40]^ Changes to coat protein sequence, temperature, or buffer conditions can also induce polymorphic behavior in protein cages.^[41]^ For example, the S37P mutation in the coat protein of MS2 results in a shift from *T* = 3 to *T* = 1 symmetry,^[42]^ and one point mutation in the normally icosahedral *Myxococcus xanthus* encapsulin was recently found to be sufficient to induce the formation of tetrahedra.^[43]^ Heating or treatment with chaotropic chemicals causes the P22 capsid, which naturally assembles into *T* = 7 icosahedral symmetry, to expand and lose its pentameric facets to generate large holes in the capsid structure.^[44]^

Non-icosahedral structures can also be formed alongside icosahedral particles. Examples include a recent study of infectious Q*β* by Zhang and co-workers, showing the presence of *T* = 4, prolate, oblate, and small prolate cages in addition to the normal *T* = 3 structure.^[45]^ The nonstandard structures each contained 12 pentamers but variable numbers of hexameric units (from 12 to 30). Each structure naturally exhibited different curvatures, presumably due to changes in flexible loops that provide variations in hexamer plasticity. We have also found that a dimeric form of the Q*β* protein (made by genetically linking the N-terminus of one Q*β* coat protein to the C-terminus of another with an AYGG tetrapeptide, similar in design to dimeric forms of MS2^[46,47]^ and PP7^[22]^) produced the same array of self-assembled structures as observed by Zhang, plus three additional assembly states [C2, C3, and tetrahedral (T)].^[48]^ Heddle and co-workers identified five distinct cages formed by VLPs assembled from the fusion of the SpyTag domain or random sequences to the C-terminus (residue 144G) of the MS2 coat protein. These structures included icosahedral *T* = 3 and *T* = 4 assemblies, one assembly with D5 symmetry, and two with D3 symmetry, all containing 12 pentamers and varying numbers of hexamers.^[49]^

PP7, like Q*β* and MS2, is a member of the *Leviviridae* family of bacteriophages, notable for their high tolerance of genetic and chemical modifications and exceptional stability under reducing conditions and high temperatures.^[19,22,23]^ The canonical *T* = 3 form of PP7, containing 180 copies of the coat protein (CP), is 28.4 nm in diameter; we designate this as the monomer (or wild-type, WT) particle. Due to the strong interaction between the CPs, the capsid can be considered as having 90 copies of the noncovalent CP dimer.^[50,51]^ Within these dimers, the C-terminus of one monomer is adjacent to the N-terminus of the other. In addition to using the AB loop of the CP monomer of PP7 and MS2 as a site of peptide insertion,^[22,52]^ Peabody and co-workers have genetically linked the C and N termini with a YG dipeptide bridge, chosen for reasons of cloning convenience, and thereby produced particles that were apparently (by low resolution methods such as size-exclusion chromatography) identical in size and shape to those self-assembled from the parent monomeric coat protein.^[5]^ We have reported such “PP7 dimer” particles using a slightly different bridging sequence—an AYGG tetrapeptide linker (also chosen for ease of cloning)—which formed *T* = 4 icosahedral cages consisting of 120 genetically linked CP dimers, as revealed by high resolution cryo electron microscopy (EM).^[23]^ In a later study, 15 amino acid libraries were added between the two glycine residues in the bridge between subunits (AYG-15mer-G).^[53]^ Beside icosahedral assemblies, approximately 40% of the particles were found by cryo-EM analysis to be non-icosahedral. However, due to the heterogeneous nature of the particle library, we were unable to further resolve distinct structures.

Here, we describe the effect of changing the oligopeptide linkage between PP7 coat proteins on the structures produced by self-assembly in the bacterial expression host. The junction was systematically varied from one to four amino acids in length, producing a remarkable diversity of structures, a consistent trend in the types of ensembles formed, and the complete absence of any capsid sequence that provided a single monodisperse product. The results add to a growing body of evidence that structural homogeneity of virus-like particles, and perhaps natural viruses as well, should not be assumed.

## 2. Results and Discussion

### 2.1. Pleomorphism of PP7 VLPs: the PP7 Capsid Population Is Not Structurally Homogeneous

**Figure 1.**
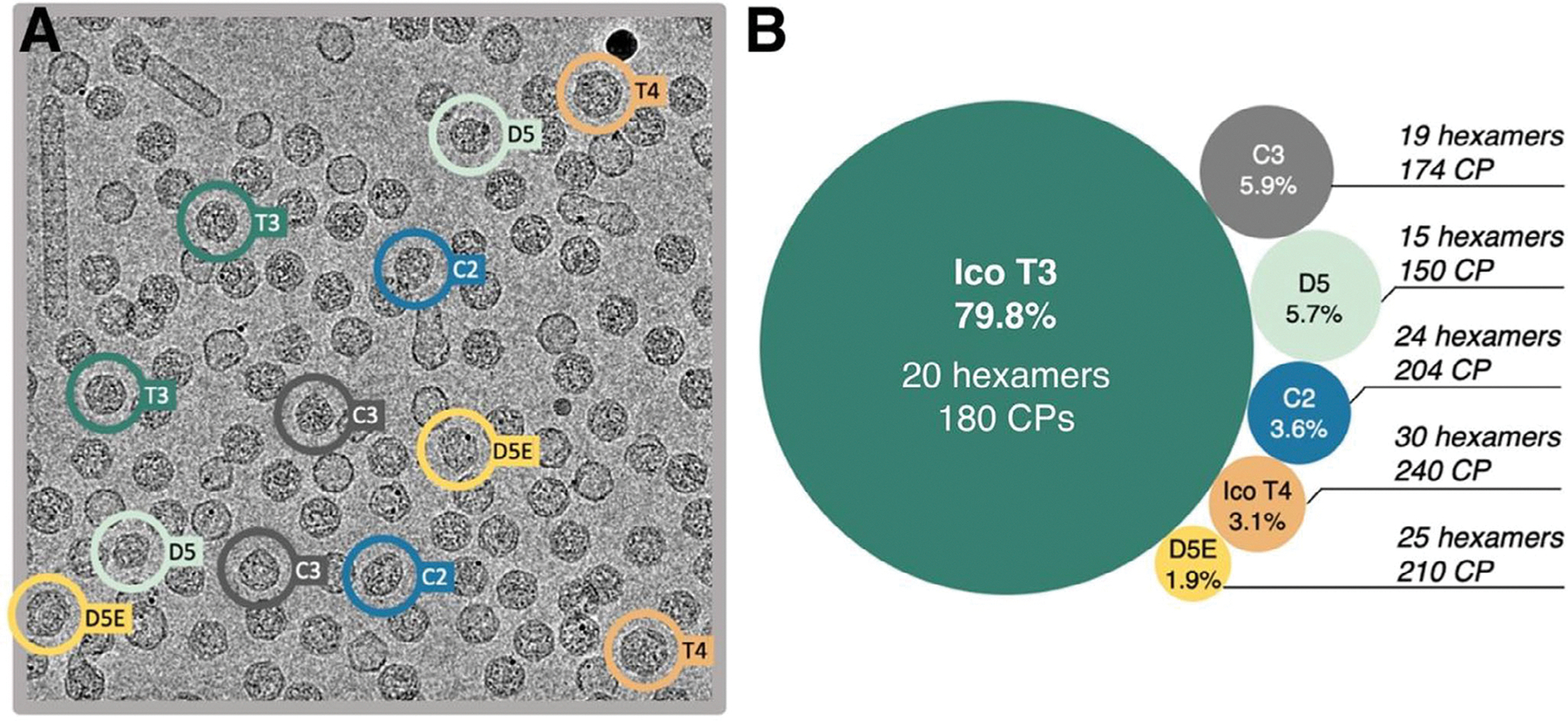
Structural features of capsid morphologies present in wild-type PP7 VLP. A) Representative micrograph featuring all six types of VLP classes, as well as tubular assemblies. B) Percent abundance and coat protein composition of different classes of VLPs: icosahedral *T* = 3, icosahedral *T* = 4 and non-icosahedral D5-, D5E-, C2-, and C3-symmetric particles. Labels indicate the symmetry group and number of coat proteins and hexamers making up the different capsid morphologies observed in PP7 WT VLPs. All cages contain 12 pentamers.

The PP7 capsid protein derived from the originally published virus sequence was previously reported to assemble into *T* = 3 icosahedral capsids.^[51,54]^ Our expression of this “parent” WTVLP and analysis by cryo-EM revealed a subset of additional assemblies with the *T* = 3 icosahedron in three independent experiments (Figure 1A). Classification of the 2D images found the same heterogeneous population in each sample: 79.8% ± 0.6% *T* = 3 particles, 3.1% ± 0.8% *T* = 4 icosahedra, and 17.1 ± 0.7% non-icosahedral structures (Figure 1B). Within the last group were a significant number of 2D structures with “potato” or “clamshell” shapes. Analysis of these samples by denaturing gel electrophoresis and mass spectrometry revealed no detectable amounts of any other protein (Figure S2, Supporting Information), implying that these unusual forms were also composed of PP7 CP.

**Figure 2.**
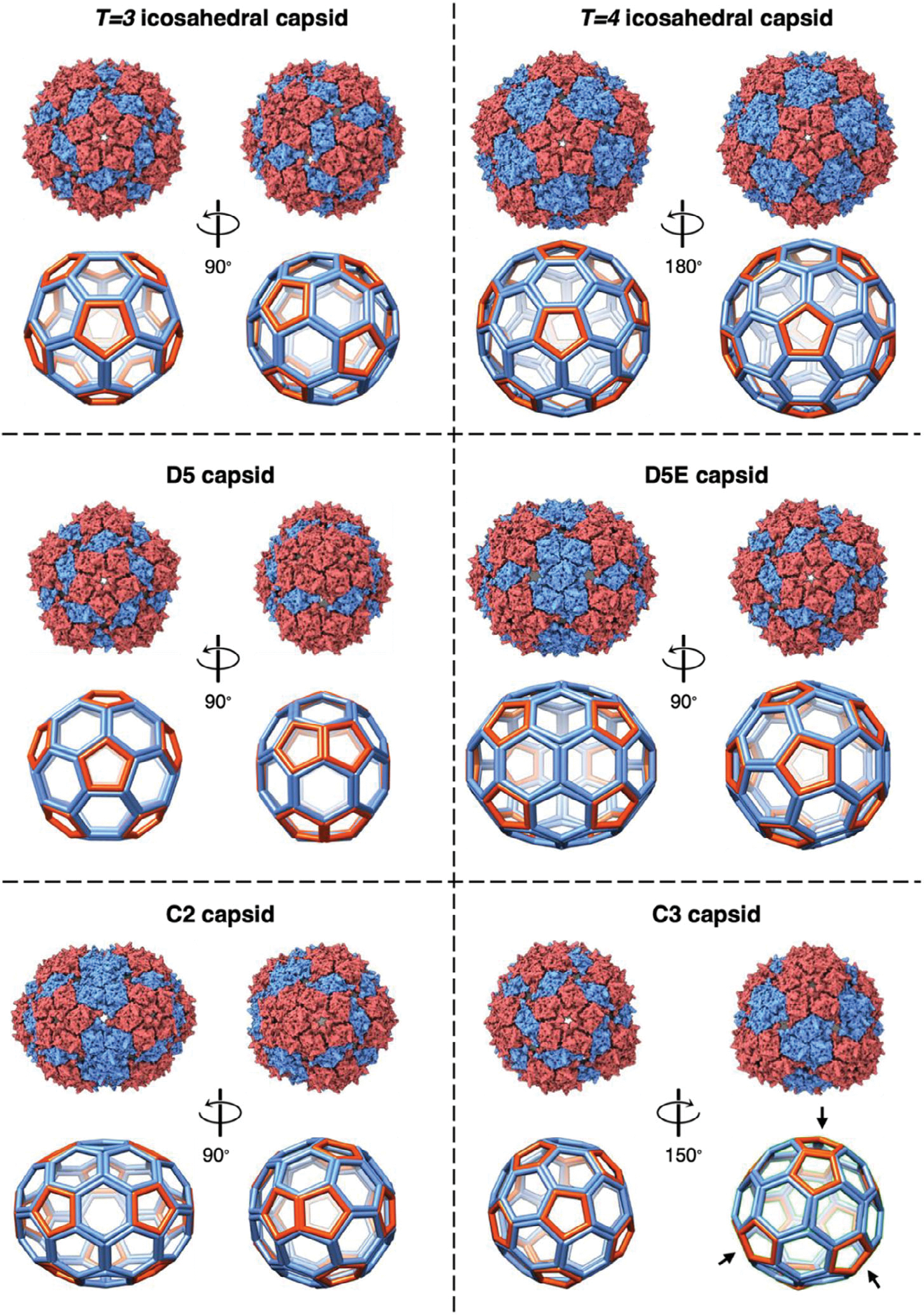
Cage structures of the spherical assembly states in the PP7 dimer VLPs, highlighting the pentameric units in red and hexameric units in blue. *T* = 3 icosahedral (top, left) and D5 (middle, left) cages having similar pentamer/hexamer geometry in the left views, while significantly different geometry upon 90° turn. D5E (middle, right) cage having an extended body compared to D5 (middle, left). C3 cage (bottom, right) with the arrows pointing at the overlapping pentamers. C2 (bottom, left) capsid with the overlapping pentamers shown at the rotated angle. Similarities to *T* = 3 (top, left) and *T* = 4 (top, right) icosahedra can be observed in the capsid. Structures deposited in EMDB, shown here, are derived from the PP7-AY-PP7 construct with resolutions listed in Table S2 (Supporting Information).

The same types of structures were observed for PP7 dimer particles; in-depth analysis of the particles formed by the AY-bridged dimeric coat protein are shown in Figure 2. 3D reconstruction of the non-icosahedral population of these particles allowed us to identify four unique capsid geometries, possessing D5, extended D5 (referred to as “D5E”), C2, and C3 symmetries. All four structures are made of 12 pentamers with varying number of hexamers. The D5 capsids are small, pentagon-shaped (clamshell) structures containing a total of 150 coat proteins. Although the frontal view of this capsid looks similar to the *T* = *3* icosahedron, both containing a pentamer (displayed in red) in the center separated from five surrounding pentamers by a dimer subunit (in blue), the side view of the D5 capsid is much more angular. Due to the dihedral symmetry, the five surrounding pentamers in the front overlap with the five pentamers in the back. This contact is better visualized through a 90° turn in Figure 2 and Movie S1 (Supporting Information). The D5E capsids are an elongated version of the D5 particles, in which the overlapping pentamers are separated by a belt of hexamers (Figure 2 and Movie S2, Supporting Information). All of these structures were found in the data sets for the parent monomer particle as well. The D5 and D5E structures here correspond to the oblate and prolate capsids observed in Q*β*, respectively.^[45]^ Similarly, D5E capsids were also reported for MS2 dimer VLPs that contained peptide insertions at the 144G position.^[49]^

The C3 capsid contains 87 copies of the PP7 dimeric CP (Figure 2) or 174 copies of the monomeric CP (Figure 1), and represents an interesting melding of structural components. Half of the particle consists of pentamers separated via dimer subunits, similar to the orientation observed in *T* = 3 icosahedra. The other half consists of three pairs of overlapping pentamers (Figure 2 shown with arrows, Movie S3, Supporting Information), with the overlapping pentamers being a characteristic of *T* = 1 icosahedra. However, these three sets of pentamers surround three hexamers, a structural property of *T* = 4 icosahedra.

The C2 particle is comprised of 102 copies of the PP7 dimeric CP, or 204 copies of the monomer, assembled into an oval shaped particle which also has elements of different icosahedral cages (Figure 2). Two overlapping pentamers provide pointed caps to the capsid, both leaning in the same direction (Movie S4, Supporting Information). One of the cap pentamers is separated from three neighboring pentamers with CP dimer units in a fashion similar to *T* = 3 icosahedral particles, and the other cap pentamer is separated from neighboring pentamers by trimers of dimers, in a similar fashion to the *T* = 4 capsid. Such an arrangement disrupts rotational symmetry along the long axis of the structure, leaving only a C2 symmetry axis.

**Figure 3.**
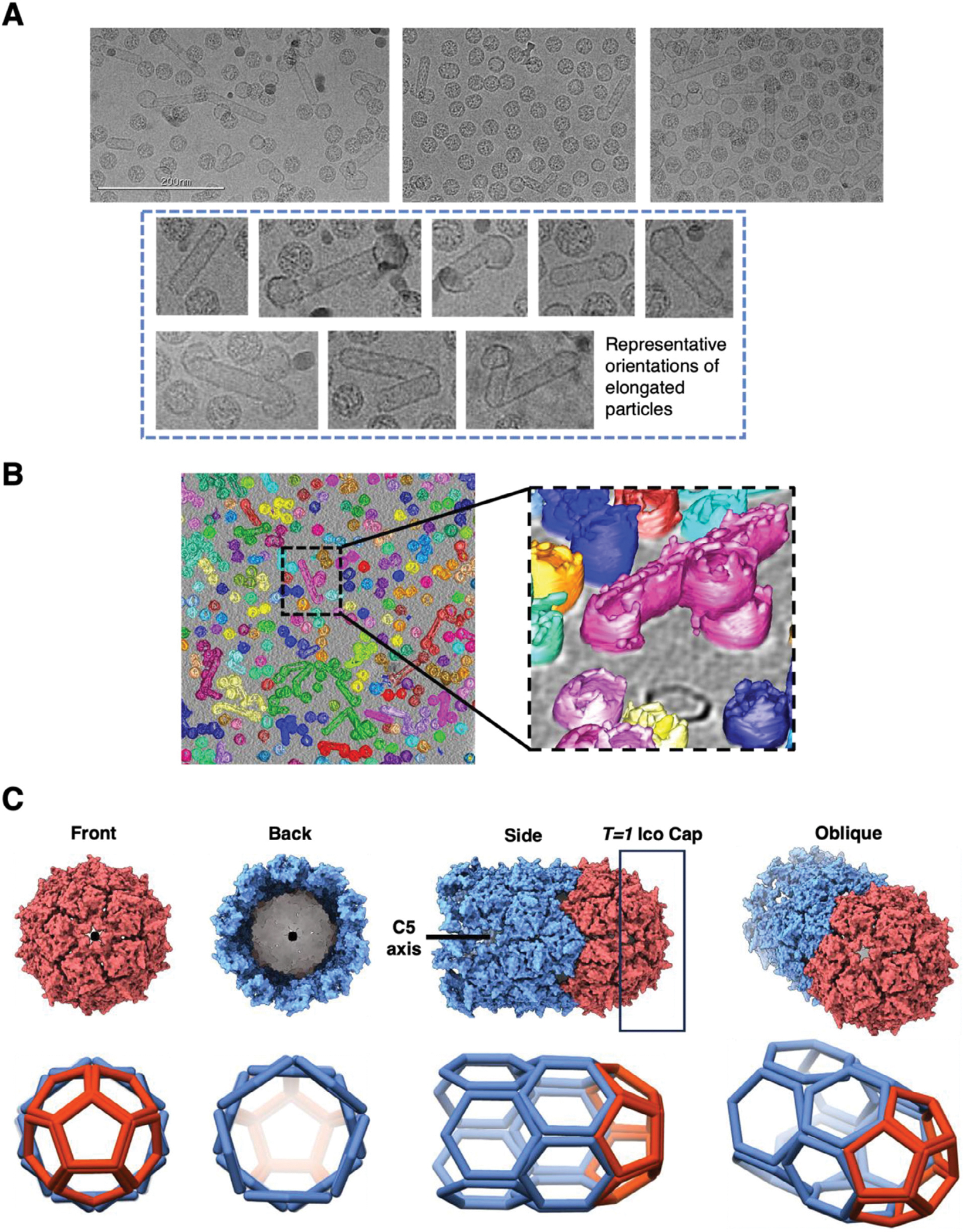
Various morphologies of rod-shaped assemblies in VLPs derived from monomeric PP7 capsid protein. A) Cryo-EM micrographs from three independently expressed, purified, and imaged PP7 VLPs, all displaying spherical and rod-shaped particles. The boxed images represent eight unique rod-shaped morphologies, with variations in the orientation of their caps and/or curvature. All images are scaled evenly. B) PP7 VLP heterogeneity observed through cryo-ET. An illustration of layers of the grid is shown in Movie S5 (Supporting Information). C) Surface and cage structures of the normal tube with a blunt end, possessing C5 symmetry.

Besides the expected and unexpected spherical assemblies, we also detected various rod-shaped particles in most PP7 VLP samples derived from both monomeric and dimeric coat proteins; these structures were especially prominent in some, but not all, WT preparations (Figure 3A). Four replicates of PP7 monomer VLPs were independently transformed, expressed, purified, and imaged. The rod-shaped assemblies were present in all four replicates; however, they differed significantly in their total population. Several factors may contribute to this variability, including fluctuations in temperature during expression, different degrees of separation of rod-shaped structures from others during purification, or differences in the location of particles in the EM grids since ice thickness can vary and can “squeeze out” larger structures. We therefore assign significance only to the appearance of rod-shaped morphologies (and aspects of their structures, as follows), but not to their relative abundance.

The variation in the cap geometry is an interesting characteristic of these rod-shaped assemblies, with “blunt” and “irregular” caps being observed at either or both ends of a tube (Figure 3A). Most of the tubular assemblies terminate with the “blunt” C5 symmetrical cap consisting of only overlapping pentamers. Such ends represent one half of a (non-existent) *T* = 1 icosahedral structure (Figure 3C). The remainder of the tube is made of repeating rings of hexamers that allows the tube to be linearly extended.

To ensure the tubular structures and the caps are indeed connected rather than an artifact created by the positioning of tubular and spherical VLPs next to each other on the micrographs, 3D information of the shape of individual structures was obtained by cryo-electron tomography (cryo-ET).^[55]^ The tomogram reconstruction, shown in Figure 3B and Movie S5 (Supporting Information), verifies that the irregular caps share a common lumen with the tube, either positioned directly centered to the body of the tube or tilting one way.

### 2.2. Morphology Is Sensitive to Linker Length and Sequence Connecting Dimer Coat Proteins

**Figure 4.**
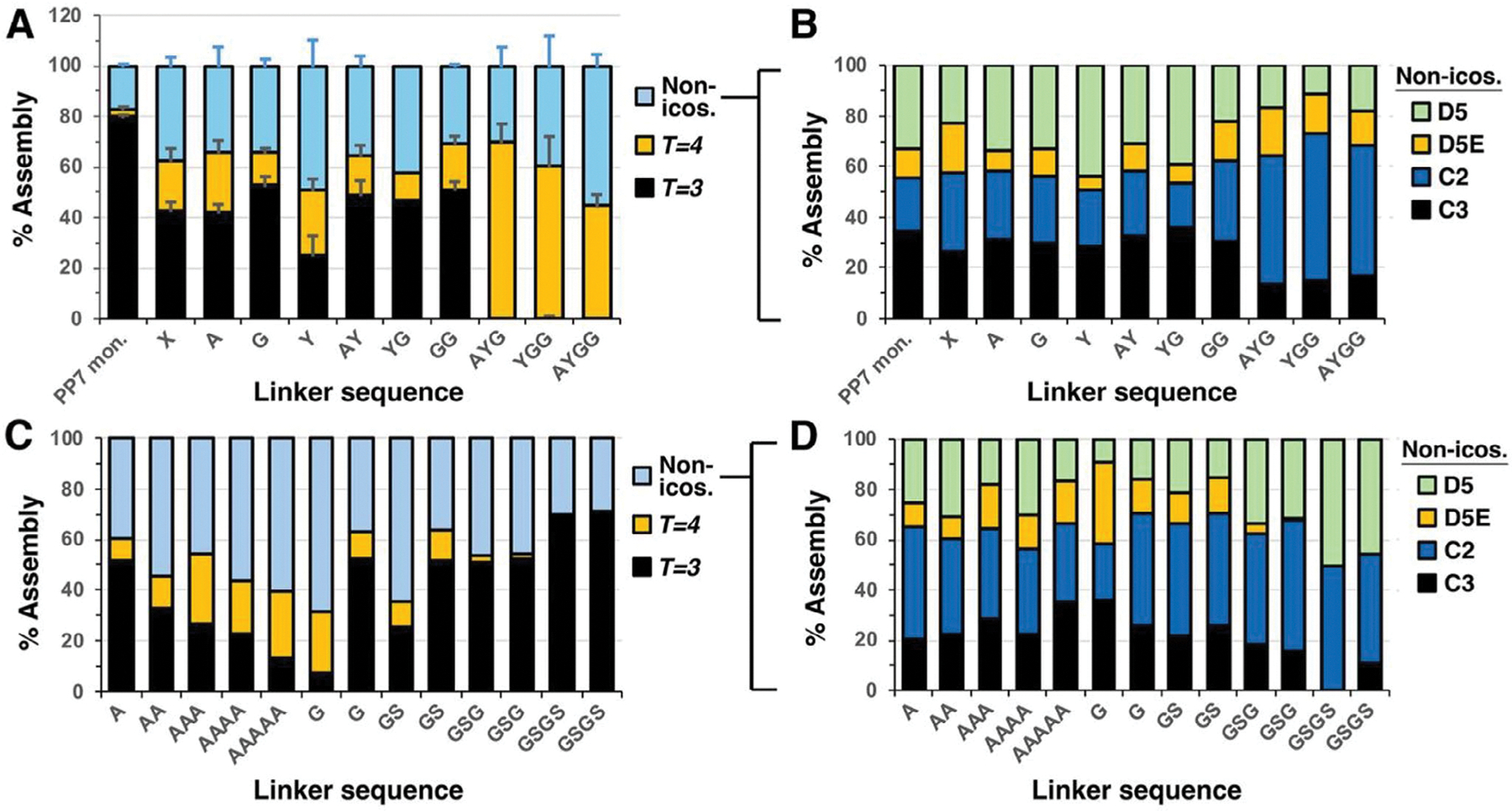
Percent composition of various assemblies in PP7 dimer variants. A,C) Percent *T* = 3, *T* = 4, and non-icosahedral structures. B,D) Breakdown of D5, D5E, C2, and C3 capsids within the non-icosahedral pool of particles in the constructs shown in panels A and C, respectively. “PP7 mon.” = VLP of the monomeric capsid protein.

Given the change between predominance of *T* = 3 capsids for the wild-type PP7 monomer VLP and the predominance of *T* = 4 particles for the AYGG-linked dimer (and for dimers with larger peptide insertions^[23,53]^), we explored the structural space formed by PP7 proteins connected with a variety of short linker lengths. Nine constructs were prepared in a first series, each encoding dimeric PP7 CPs connected by the following linkers, ranging from zero to three amino acids derived from the original AYGG linker: none (X), A, G, Y, AY, YG, GG, AYG, YGG, and AYGG. Triplicates of each construct were recombinantly expressed from separate bacterial colonies, purified, and analyzed by cryo-EM, as discussed in Materials & Methods, with the results summarized in Figure 4A,B.

These PP7 dimer constructs, linked by zero, one, and two amino acids, assembled with a mixture of substantial amounts of *T* = 3 and non-icosahedral structures (Figure 4A). Among these constructs, six out of seven favored *T* = 3 icosahedra, but the assembly landscape was apparently dynamic enough to allow for 10%–30% *T* = 4 particles as well. The tyrosine-linked structure was the exception, forming roughly equal amounts of icosahedral and non-icosahedral assemblies, with approximately equal amounts of *T* = 3 and *T* = 4 in the former category. Remarkably, the introduction of just one more amino acid to the linker fully abolished the *T* = 3 structure. Reproducibility of morphological distributions among particles isolated from different host bacterial colonies was fairly good, as shown by the standard deviations in Figure 4A.

These observations were found to be sequence-dependent, illustrated by the preparation of two additional series of dimeric particles: one containing increasing numbers (1–5) of inserted alanine residues and the other containing the insertion of G, GS, GSG, and GSGS (Figure 4C,D and Figures S5 and S6, Supporting Information). The latter series was repeated independently (including *E. coli* transformation with plasmid). Thus, the particles with A_1_–A_5_ inserts exhibited a gradual, rather than a sharp, decrease in *T* = 3 icosahedra, with a concomitant increase in non-icosahedral particles rather than *T* = 4 capsids. Furthermore, the G/S series showed the opposite trend with insertion length, increasing the proportion of *T* =3 structures and almost completely eliminating *T* = 4 particles in the GSG and GSGS inserts. These two constructs were highly reproducible in their morphological distribution, but the G- and GS-bridged dimers were not when starting from separate bacterial cell transformations (one of the G-insert preparations closely matching the one previously made and shown in Figure 4A, but the other not).

Among non-icosahedral particles, the C2 and D5 classes were consistently represented in all preparations (Figure 4B,D), and C3 particles were quite abundant in many, but not all, samples. The D5E structure was the least abundant, again on a consistent basis. However, no trend could be discerned that connected non-icosahedral structural distributions with the relative amounts of *T* = 3 and *T* = 4 particles formed. For example, in the AYGG series, C2 structures seemed to be positively correlated with the formation of *T* = 4 icosahedra, but no such relationship was observed among the other series.

We hasten to note that precise quantitative reproducibility in statistical distributions of this kind by cryoEM is often difficult. In general, protein samples behave differently during vitrification, and some can be under- or overrepresented in thin ice sheets because of molecular size, shape, and surface properties, as well as other variables such as the choice of support film, grid cleaning parameters, and ice thickness. However, we expect the analyses of these particles to be more consistent than in many other cases, since they are all somewhat similar in size and gross shape, their surface properties are nearly identical, and care was taken to use the same settings and grid type in the preparation of cryo-EM samples.

## 3. Conclusion

Capsid polymorphism is a well-recognized phenomenon in natural viruses and has long been observed in “artificial” structures such as virus-like particles.^[56,57]^ VLPs, however, have often been assumed to be monodisperse in structure; we have made this kind of assertion ourselves.^[25,58]^ A related idea is that the nature of icosahedra, as the most efficient 3D closed shape in surface/volume ratio, provides an energetic advantage and leads to monodispersity. Here we showed that despite being capable of self-assembly into icosahedral particles, a sizable number of PP7-WT particles form symmetric closed non-icosahedral cages. We also demonstrated that very small changes in a peptide sequence linking two coat proteins induced dramatic changes in the distribution of these structures, and that these changes are of a very different nature for different amino acids used in the linkage (Figure 4). It therefore appears that these VLP morphological distributions are stochastic in nature, consistent with the expectation that the assembly pathways toward each of the observed structures are close in energy and may therefore lead to significant variations in the final distributions of structures, even without the certain existence of variations in conditions (protein expression rates, concentrations of protein and nucleic acid, presence of unknown host factors, etc.) that can affect self-assembly. [It is also possible for the observed structures to be kinetically-trapped intermediates, but we think this unlikely: the temperatures or extremes of pH required to affect even partial disassembly of these particles are far outside what is accessible under biological conditions.^[59,60]^ This hypothesis, however, remains to be conclusively tested.]

It should also be noted that the packaged nucleic acids in natural viruses have a profound impact on the self-assembly process,^[61]^ altering or directing the distribution of structures.^[62–66]^ Indeed, the initiation of assembly by formation of pentamers or other substructures is often driven by specific interactions of CP with the genetic material,^[3,4]^ and such interactions play important roles in viral evolution and fitness.^[67]^

Nevertheless, in the absence of co-evolved genetic cargo, it is clear that virus-like self-assembling subunit building blocks of the type studied here are not so precisely evolved as to be restricted to the formation of only one structure. Some plasticity in the self-assembly function of natural viruses—perhaps aiding in tolerance of mutational variations^[68–70]^—could be helpful if multiple different forms can fulfill the key functions of genome packaging and delivery required of the system. Such pleomorphism can also occur in other self-assembling protein cages: we have recently described wild-type and genetically modified bacterial (*Myxococcus xanthus*) encapsulins that can assemble into seven different assembly states, five of which are non-icosahedral. The percentage of the non-icosahedral assemblies rises when foreign sequences are incorporated into loops in the wild-type sequence.^[71]^

From a chemical perspective, the power of subunit proteins to noncovalently interact with each other in five- and six-fold symmetry combinations—crucially, without diverting substantially to uncontrolled aggregation—is the key property. Proteins designed in the laboratory to self-assemble into closed structures also often exhibit pleomorphism. Examples include structures from Yeates and co-workers,^[72]^ laboratory evolution of the *Aquifex aeolicus* lumazine synthase catalytic nanocage by Hilvert et al., in which structural pleomorphism was found to be a feature in early rounds of selection,^[73]^ and engineered fused coiled-coil domain proteins from Marsh and co-workers.^[74–76]^

From the molecular engineer’s perspective, these and related findings reinforce the need to be comfortable with ensembles of particles. We already are unable to avoid such mixtures in the chemical modification of protein nanoparticles, for which we characterize average numbers of attachments, understanding that each particle differs from most others in the exact number and arrangement of the added moieties. With pleomorphism such as that observed here for PP7 dimers, we make ensembles even when the positions and numbers of chemical modifications are precisely controlled by genetic programming, such as by the use of unnatural amino acids.^[77,78]^ While scaffolds having dramatic differences in shape, such as tubes versus roughly spherical particles, may be expected to exhibit greater differences in properties,^[79]^ we anticipate that properties and applications such as polyvalent binding and immunogenicity are unlikely to be very different for particles of *T* = 3, C3, or D5 symmetry, for example. However, this will have to be an assumption in many cases, as it will be quite difficult to either force truly monodisperse assembly or separate one structural form away from the others on a practical scale.

## Supplementary Material

Supporting Information

SI movie #1

SI movie #2

SI movie #3

SI movie #4

SI movie #5

Supporting Information

Supporting Information is available from the Wiley Online Library or from the author.

## Data Availability

All cryo-EM maps were deposited to EMDB (https://www.ebi.ac.uk/emdb/) with the following access codes and deposition titles: EMD-44737: WT *Pseudomonas* phage PP7 capped tube VLP; EMD-44743: WT *Pseudomonas* phage PP7 open tube VLP; EMD-44767: C2 cage of PP7-AY-PP7: *Pseudomonas* phage PP7 coat protein dimer; EMD-44768: C3 cage of PP7-AY-PP7: *Pseudomonas* phage PP7 coat protein dimer; EMD-44773: D5 cage of PP7-AY-PP7: *Pseudomonas* phage PP7 coat protein dimer; EMD-44782: D5E cage of PP7-AY-PP7: *Pseudomonas* phage PP7 coat protein dimer; EMD-44783: Icosahedral *T* = 3 cage of PP7-AY-PP7: *Pseudomonas* phage PP7 coat protein dimer; EMD-44788: Icosahedral *T* = 4 cage of PP7-AY-PP7: *Pseudomonas* phage PP7 coat protein dimer.
