# Supplementary material for: Pleomorphism in Wild-Type and Engineered PP7 Virus-Like Particles
Source: Small. Author manuscript; Available in PMC 2026 Jan 17. (PMC12710124; doi:10.1002/smll.202506285)
Supplement: Supporting Information [file NIHMS2130217-supplement-Supporting_Information.pdf]

Supporting Information

Pleomorphism in wild-type and engineered PP7 virus-like particles

Parisa Keshavarz-Joud, Matthew C. Jenkins, Tahiti Dutta, Liangjun Zhao, Carolina Hernandez, Daija Bobe, Mohammadreza Paraan, M.G. Finn, Mykhailo Kopylov**

1. Materials & Methods

Plasmids

In all constructs, we used the pCDF1b vector harboring PP7 coding sequences under a T7 promoter. Our previously reported PP7 dimer expression plasmid containing the AYGG linker sequence[1] was modified to either shorten the original AYGG linker or to alter the linker to the desired sequence, as detailed below, using New England Biolabs (NEB) Q5 Site-Directed Mutagenesis Kits following the manufacturer's protocol.

Expression and purification

BL21 (DE3) chemically competent *E. coli* cells (NEB) were transformed with plasmids harboring the PP7 VLPs according to manufacturer's protocol. The transformed cells were grown in 2YT media supplemented with 50 µg/mL of streptomycin. A 50 mL starter culture was grown at 37 °C with shaking (200 rpm) for 16-18 hours, and then used to inoculate a 500 mL culture at 1:50 ratio. The culture was incubated at 37 °C with shaking (200 rpm) until an OD_{600} value of 0.8–1.0 was reached. Protein expression was induced with 1 mM isopropyl β-D-1-thiogalactopyranoside (IPTG), and the expression culture was maintained at room temperature with shaking (200 rpm) overnight. Cells were harvested by centrifugation at 8,000 rpm for 15 minutes. The PP7 VLP variants were purified as previously reported.[2] Briefly, cells harvested from 250 mL of cell culture were resuspended in 40 mL of potassium phosphate buffer (0.1M, pH 7.5) and sonicated at 50–60% amplitude for 10-15 minutes with 5-second bursts separated by 5-second intervals using a QSonica Q500 immersion sonicator. The clarified cell lysate supernatant was incubated with a final concentration of 26.5% ammonium sulfate (w/v) for 2 hours to precipitate out the PP7 VLPs. The mixture was centrifuged for 10 minutes at 13,000 rpm and the pellet was resuspended in potassium phosphate buffer (0.1M, pH 7.5). Residual lipids and membrane proteins were removed by 1:1 *n*-butanol:chloroform organic extraction. The top aqueous phase containing VLPs was layered onto a 10-40% sucrose gradient cushion and centrifuged at 28,000 rpm for 4 hours at 4 °C. The VLP fraction, visualized as a blue band under white light, was collected and pelleted out by ultracentrifugation at 68,000 rpm for 2 hours at 4 °C. The VLP pellet was slowly dissolved in potassium phosphate buffer (0.1M, pH 7.5) overnight on a shaking platform at 4 °C.

VLP Characterization

The concentration of PP7 protein constructs were determined using Coomassie Plus Protein Reagent (Pierce) with bovine serum albumin (BSA) as standard. Coat protein molecular weights were assessed by LC-MS (ESI–ToF, Agilent). VLP samples were first denatured by incubation of 10 μL of 1.0 mg/mL VLP solution with a mixture of 2.5 μL of 1M DTT and 2.5 μL of 1M urea for 5 minutes at room temperature) followed by dilution with 90 μL water and centrifugation at 13,000 rpm for 2 minutes to remove aggregates. 1–2 μL of each sample (corresponding to 50–100 ng VLP) were introduced into the mass analyzer after passage through a C3 reversed-phase HPLC column.

CryoEM sample preparation, data collection and data processing

R1.2/1.3 quantifoil (carbon foil) holey grids (EMS, 300 mesh) were plasma-cleaned using Solarus inGatan Plasma System in an oxygen-argon environment at 15 W power. VLPs were diluted to 1 mg/mL, and an aliquot of 3 µL of sample was applied to the foil side of the grid. Grids were plunge-frozen into liquid ethane on a Vitrobot Mark IV automatic plunge freezer (ThermoFisher Scientific) after blotting for 3 s with blot force of 0. Frozen grids were clipped in AutoGrid rings and stored in liquid nitrogen until data collection.

Single-particle data were acquired on Krios G2 cryo-TEM equipped with Gatan K3 camera and BioQuantum energy filter (NYSBC Krios 1). The acquisition parameters were as follows: nominal magnification 81,000x, pixel size 1.083 Å, exposure time of 2000 ms, total dose of ~50 e-/Å2 per image in movie mode with 40 frames per movie and random nominal defocus ranging from -1 μm to -2 μm. For each sample a dataset consisting of at ~1,000 images were collected to ensure that at least 100,000 particles were available for 3D classification and reconstruction. The datasets that were used for final reconstructions are summarized in Table S1, taken from the third replicate.

Single-particle data were analyzed in cryoSPARC 4.2.1. Movies were imported, patch motion-corrected and patch CTF-estimated with default settings. Blob picker was used on a 100-micrograph subset with particle diameter set to 250-300 Å and maximum number of local maxima set to 1000. A total of 15,254 particles were extracted with a box size of 512 and 2D-classified with default settings. Sharp and well-centered templates were selected to be used as templates for full-dataset particle picking. Sample PP7-A-PP7 was used for initial identification of assembly variants.

Multiple rounds of 2D and 3D classification were used to identify different cage assembly variants. A representative processing workflow (for the PP7-A-PP7 dataset) is depicted in Figure S1. The particle stack containing 162,818 particles was separated into three sub-stacks using 2D classification with 50 classes. Icosahedral T=3 (smaller) and T=4 (larger) classes were identified by visual inspection and separated into individual sub-stacks with 60,750 and 29,506 particles respectively. All non-icosahedral particles were separated into a third sub-stack containing 66,810 particles and the remaining particles from "junk" classes were discarded.

Particles from the T=3 icosahedral sub-stack were used to generate *ab initio* reconstructions with two classes and C1 symmetry. The resulting *ab initio* classes were independently refined with C1 symmetry and visually inspected to identify symmetry, revealing a previously uncharacterized C3-symmetric particle class with 12,245 particles and a T=3 icosahedral class with 48,505 particles. Each set of particles was further refined using

homogeneous refinement with corresponding symmetries applied, producing T=3 and C3 reference maps.

Particles from the T=4 sub-stack were processed in the same way as the T=3 sub-stack. The *ab initio* reconstruction classes were similar, both showing icosahedral symmetry, suggesting that the T=4 sub-stack contained only one type of the particles. All particles were further refined using homogeneous refinement with icosahedral symmetry applied, producing a T=4 reference map.

Particles from the non-icosahedral sub-stack were first classified using *ab initio* reconstruction with two classes and C1 symmetry. The resulting *ab initio* classes were independently refined with C1 symmetry and visually inspected to identify symmetry, revealing a previously uncharacterized D5-symmetric particle class with 15,724 particles and a poorly resolved, class with 51,806 particles. The D5-symmetric particle class was further refined using homogeneous refinement with D5 symmetry applied, producing a D5 reference map. The poorly resolved class was classified again using *ab initio* reconstruction with two classes, resulting in two classes – one with C2 symmetry and another class with D5 symmetry. This D5-symmetric class contained larger particles than the previously identified D5 reference. Both sets of particles were further refined using homogeneous refinement with corresponding symmetries applied, producing C2 and D5e (D5 extended) reference maps.

The resulting six constructs were used as starting models for heterogeneous refinement of all datasets to estimate the particle numbers for each class. The final reconstructed maps were projected as 2D templates and compared with the results of 2D classification of each class to ensure that all 2D classes were accounted for. For an additional verification, particles from the final reconstructions of each class were subjected to 3D classification using *ab initio* reconstruction with two or three classes and C1 symmetry. For all six classes this only produced maps similar to the originally assigned class, suggesting that all stacks contained predominantly one type of particles per stack.

The processing for all other datasets was streamlined by omitting ab initio classifications (Figure S1 – dashed box). Specifically, all preprocessing steps, including motion correction, CTF estimation, particle picking, particle extraction and 2D classification were the same across all datasets. For each dataset initial 2D classes were visually inspected to remove junk particles. The resulting particle stack was used directly as an input for heterogeneous refinement with six classes, using the six reference maps identified for PP7-A-PP7 dataset. The same verification workflow as for PP7-A-PP7 was applied to all datasets, with no new assembly variants identified. The highest-resolution maps were obtained from processing of the PP7-AY-PP7 dataset.

Final reconstructed maps were projected as 2D templates and compared with the results of 2D classification to ensure that all 2D classes were accounted for. Resulting six constructs were used as starting models for heterogeneous refinement of all datasets to estimate the particle numbers for each class. Highest-resolution maps were obtained from 3D refinement with symmetry applied of classes from PP7-AY-PP7. Maps from these reconstructions have been deposited to EMDB (Table S2).

Table S1. Cryo-EM data analysis for representative sets of replicates, showing the number of micrograph images and curated particles for each construct.

Construct	Images	Curated particles	Construct	Images	Curated particles
PP7-A-PP7	1,015	157,066	PP7-A-PP7 (repeat)	797	85,753
PP7-AYG-PP7	779	105,055	PP7-AA-PP7	833	100,412
PP7-AY-PP7	3,500	252,316	PP7-AAA-PP7	1,035	122,989
PP7-X-PP7	1,078	170,354	PP7-AAAA-PP7	2,751	95,815
PP7	1,522	156,343	PP7-AAAAA-PP7	863	91,290
PP7-AYGG-PP7	1,528	151,897	PP7-G-PP7	1011	159,136
PP7-YGG-PP7	1,178	108,366	PP7-GS-PP7	1677	270,673
PP7-Y-PP7	1,372	194,123	PP7-GSG-PP7	1399	211,543
PP7-GG-PP7	814	112,002	PP7-GSGS-PP7	528	75087
PP7-G-PP7	1,003	150,854			
PP7-YG-PP7	1,149	206,226			

Table S2. Refinement statistics obtained from the PP7-AY-PP7 construct giving the resolutions listed, which were the best obtained for each structure.

Symmetry	Particles	GSFSC resolution, Å	b-factor	EMDB
Icosahedral T=3	118,871	3.05	159.8	EMDB-44783
Icosahedral T=4	36,959	3.23	143.5	EMDB-44788
D5	29,737	3.62	85.3	EMDB-44773
D5E	10,515	3.74	66.5	EMDB-44782
C2	30,148	4.40	56.6	EMDB-44767
C3	31,329	3.82	60.2	EMDB-44768

Cryo-electron tomography data collection and processing

Tilt series were acquired on the same cryo-EM grids and microscope using SerialEM 4.0 beta. The tilt series scheme was dose-symmetric with a tilt range of -60 to +60, tilt step of 3 degrees and a per-tilt dose of 3 e/Å^2 spread out over 10 frames. The movie frames were imported into Warp[3] for further processing.

Tilt series were motion corrected using Warp and the tilt series stacks were generated. Tilt series alignment and tomogram reconstructions were done using AreTomo.[4] These tomograms were segmented using TomosegmemTV.[5] The cryo-ET figure and movie were generated in IMOD.[6]

2. Coding sequences of PP7 variants

Cyan highlight: PP7 N-terminal coat protein
Grey highlight: PP7 C-terminal coat protein
Underlined & bold: linker sequence

WT PP7:
ATGTCGAAAACCATCGTCCTGTCCGTGGGCGAAGCAACCCGCACCCTGACCGAAATCCAATCTA
CCGCAGACCGCCAAATCTTTGAAGAAAAAGTGGGTCCGCTGGTCGGTCGTCTGCGTCTGACCGC
CTCTCTGCGTCAGAACGGCGCGAAAACGGCCTATCGCGTCAATCTGAAACTGGATCAAGCAGAC
GTGGTTGATTGCAGCACCTCTGTTTGTGGTGAACTGCCGAAAGTGCGTTATACGCAGGTTTGGTC
ACATGACGTCACCATTGTGGCAAACTCGACGGAAGCTAGTCGCAAATCCCTGTACGATCTGACCA
AATCCCTGGTGGCGACCTCTCAAGTGGAAGACCTGGTGGTGAACCTGGTGCCGCTGGGCCGCT
AA

PP7-AYGG-PP7:
ATGAGCAAAACCATTGTTCTGAGCGTGGGTGAAGCGACCCGTACCCTGACCGAAATCCAGAGCA
CCGCTGACCGTCAAATTTTTGAGGAAAAAGTGGGTCCGCTGGTTGGCCGTCTGCGTCTGACCGC
GAGCCTGCGTCAGAACGGTGCGAAGACCGCGTACCGTGTGAACCTGAAACTGGACCAAGCGGA
TGTGGTTGATTGCAGCACCAGCGTTTGCGGCGAGCTGCCGAAAGTGCGTTACACCCAGGTTTGG
AGCCACGATGTGACCATCGTTGCGAACAGCACCGAAGCGAGCCGTAAGAGCCTGTATGATCTGA
CCAAAAGCCTGGTGGCGACCAGCCAAGTTGAGGACCTGGTGGTTAATCTGGTACCACTTGGTCG
C**GCATATGGCGGT**TCGAAAACAATTGTCCTGTCCGTGGGCGAAGCAACCCGCACCCTGACCGAA
ATCCAATCTACCGCAGACCGCCAAATCTTTGAAGAAAAAGTGGGTCCGCTGGTCGGTCGTCTGC
GTCTGACCGCCTCTCTGCGTCAGAACGGCGCGAAAACGGCCTATCGCGTCAATCTGAAACTGGA
TCAAGCAGACGTGGTTGATTGCAGCACCTCTGTTTGTGGTGAACTGCCGAAAGTGCGTTATACGC
AGGTTTGGTCACATGACGTCACCATTGTGGCAAACTCGACGGAAGCTAGTCGCAAATCCCTGTAC
GATCTGACCAAATCCCTGGTGGCGACCTCTCAAGTGGAAGACCTGGTGGTGAACCTGGTGCCGC
TGGGCCGCTAA

PP7-X-PP7:
ATGAGCAAAACCATTGTTCTGAGCGTGGGTGAAGCGACCCGTACCCTGACCGAAATCCAGAGCA
CCGCTGACCGTCAAATTTTTGAGGAAAAAGTGGGTCCGCTGGTTGGCCGTCTGCGTCTGACCGC
GAGCCTGCGTCAGAACGGTGCGAAGACCGCGTACCGTGTGAACCTGAAACTGGACCAAGCGGA
TGTGGTTGATTGCAGCACCAGCGTTTGCGGCGAGCTGCCGAAAGTGCGTTACACCCAGGTTTGG
AGCCACGATGTGACCATCGTTGCGAACAGCACCGAAGCGAGCCGTAAGAGCCTGTATGATCTGA
CCAAAAGCCTGGTGGCGACCAGCCAAGTTGAGGACCTGGTGGTTAATCTGGTACCACTTGGTCG
CTCGAAAACAATTGTCCTGTCCGTGGGCGAAGCAACCCGCACCCTGACCGAAATCCAATCTACC
GCAGACCGCCAAATCTTTGAAGAAAAAGTGGGTCCGCTGGTCGGTCGTCTGCGTCTGACCGCCT
CTCTGCGTCAGAACGGCGCGAAAACGGCCTATCGCGTCAATCTGAAACTGGATCAAGCAGACGT
GGTTGATTGCAGCACCTCTGTTTGTGGTGAACTGCCGAAAGTGCGTTATACGCAGGTTTGGTCAC
ATGACGTCACCATTGTGGCAAACTCGACGGAAGCTAGTCGCAAATCCCTGTACGATCTGACCAAA
TCCCTGGTGGCGACCTCTCAAGTGGAAGACCTGGTGGTGAACCTGGTGCCGCTGGGCCGCTAA

PP7-A-PP7:
ATGAGCAAAACCATTGTTCTGAGCGTGGGTGAAGCGACCCGTACCCTGACCGAAATCCAGAGCA
CCGCTGACCGTCAAATTTTTGAGGAAAAAGTGGGTCCGCTGGTTGGCCGTCTGCGTCTGACCGC
GAGCCTGCGTCAGAACGGTGCGAAGACCGCGTACCGTGTGAACCTGAAACTGGACCAAGCGGA
TGTGGTTGATTGCAGCACCAGCGTTTGCGGCGAGCTGCCGAAAGTGCGTTACACCCAGGTTTGG
AGCCACGATGTGACCATCGTTGCGAACAGCACCGAAGCGAGCCGTAAGAGCCTGTATGATCTGA
CCAAAAGCCTGGTGGCGACCAGCCAAGTTGAGGACCTGGTGGTTAATCTGGTACCACTTGGTCG
C**GCA**TCGAAAACAATTGTCCTGTCCGTGGGCGAAGCAACCCGCACCCTGACCGAAATCCAATCT
ACCGCAGACCGCCAAATCTTTGAAGAAAAAGTGGGTCCGCTGGTCGGTCGTCTGCGTCTGACCG
CCTCTCTGCGTCAGAACGGCGCGAAAACGGCCTATCGCGTCAATCTGAAACTGGATCAAGCAGA
CGTGGTTGATTGCAGCACCTCTGTTTGTGGTGAACTGCCGAAAGTGCGTTATACGCAGGTTTGGT
CACATGACGTCACCATTGTGGCAAACTCGACGGAAGCTAGTCGCAAATCCCTGTACGATCTGACC

AAATCCCTGGTGGCGACCTCTCAAGTGGAAGACCTGGTGGTGAACCTGGTGCCGCTGGGCCGC
TAA

PP7-Y-PP7:
ATGAGCAAAACCATTGTTCTGAGCGTGGGTGAAGCGACCCGTACCCTGACCGAAATCCAGAGCA
CCGCTGACCGTCAAATTTTTGAGGAAAAAGTGGGTCCGCTGGTTGGCCGTCTGCGTCTGACCGC
GAGCCTGCGTCAGAACGGTGCGAAGACCGCGTACCGTGTGAACCTGAAACTGGACCAAGCGGA
TGTGGTTGATTGCAGCACCAGCGTTTGCGGCGAGCTGCCGAAAGTGCGTTACACCCAGGTTTGG
AGCCACGATGTGACCATCGTTGCGAACAGCACCGAAGCGAGCCGTAAGAGCCTGTATGATCTGA
CCAAAAGCCTGGTGGCGACCAGCCAAGTTGAGGACCTGGTGGTTAATCTGGTACCACTTGGTCG
C**TAT**TCGAAAACAATTGTCCTGTCCGTGGGCGAAGCAACCCGCACCCTGACCGAAATCCAATCTA
CCGCAGACCGCCAAATCTTTGAAGAAAAAGTGGGTCCGCTGGTCGGTCGTCTGCGTCTGACCGC
CTCTCTGCGTCAGAACGGCGCGAAAACGGCCTATCGCGTCAATCTGAAACTGGATCAAGCAGAC
GTGGTTGATTGCAGCACCTCTGTTTGTGGTGAACTGCCGAAAGTGCGTTATACGCAGGTTTGGTC
ACATGACGTCACCATTGTGGCAAACTCGACGGAAGCTAGTCGCAAATCCCTGTACGATCTGACCA
AATCCCTGGTGGCGACCTCTCAAGTGGAAGACCTGGTGGTGAACCTGGTGCCGCTGGGCCGCT
AA

PP7-G-PP7:
ATGAGCAAAACCATTGTTCTGAGCGTGGGTGAAGCGACCCGTACCCTGACCGAAATCCAGAGCA
CCGCTGACCGTCAAATTTTTGAGGAAAAAGTGGGTCCGCTGGTTGGCCGTCTGCGTCTGACCGC
GAGCCTGCGTCAGAACGGTGCGAAGACCGCGTACCGTGTGAACCTGAAACTGGACCAAGCGGA
TGTGGTTGATTGCAGCACCAGCGTTTGCGGCGAGCTGCCGAAAGTGCGTTACACCCAGGTTTGG
AGCCACGATGTGACCATCGTTGCGAACAGCACCGAAGCGAGCCGTAAGAGCCTGTATGATCTGA
CCAAAAGCCTGGTGGCGACCAGCCAAGTTGAGGACCTGGTGGTTAATCTGGTACCACTTGGTCG
C**GGT**TCGAAAACAATTGTCCTGTCCGTGGGCGAAGCAACCCGCACCCTGACCGAAATCCAATCT
ACCGCAGACCGCCAAATCTTTGAAGAAAAAGTGGGTCCGCTGGTCGGTCGTCTGCGTCTGACCG
CCTCTCTGCGTCAGAACGGCGCGAAAACGGCCTATCGCGTCAATCTGAAACTGGATCAAGCAGA
CGTGGTTGATTGCAGCACCTCTGTTTGTGGTGAACTGCCGAAAGTGCGTTATACGCAGGTTTGGT
CACATGACGTCACCATTGTGGCAAACTCGACGGAAGCTAGTCGCAAATCCCTGTACGATCTGACC
AAATCCCTGGTGGCGACCTCTCAAGTGGAAGACCTGGTGGTGAACCTGGTGCCGCTGGGCCGC
TAA

PP7-AY-PP7:
ATGAGCAAAACCATTGTTCTGAGCGTGGGTGAAGCGACCCGTACCCTGACCGAAATCCAGAGCA
CCGCTGACCGTCAAATTTTTGAGGAAAAAGTGGGTCCGCTGGTTGGCCGTCTGCGTCTGACCGC
GAGCCTGCGTCAGAACGGTGCGAAGACCGCGTACCGTGTGAACCTGAAACTGGACCAAGCGGA
TGTGGTTGATTGCAGCACCAGCGTTTGCGGCGAGCTGCCGAAAGTGCGTTACACCCAGGTTTGG
AGCCACGATGTGACCATCGTTGCGAACAGCACCGAAGCGAGCCGTAAGAGCCTGTATGATCTGA
CCAAAAGCCTGGTGGCGACCAGCCAAGTTGAGGACCTGGTGGTTAATCTGGTACCACTTGGTCG
C**GCATAT**TCGAAAACAATTGTCCTGTCCGTGGGCGAAGCAACCCGCACCCTGACCGAAATCCAA
TCTACCGCAGACCGCCAAATCTTTGAAGAAAAAGTGGGTCCGCTGGTCGGTCGTCTGCGTCTGA
CCGCCTCTCTGCGTCAGAACGGCGCGAAAACGGCCTATCGCGTCAATCTGAAACTGGATCAAGC
AGACGTGGTTGATTGCAGCACCTCTGTTTGTGGTGAACTGCCGAAAGTGCGTTATACGCAGGTTT
GGTCACATGACGTCACCATTGTGGCAAACTCGACGGAAGCTAGTCGCAAATCCCTGTACGATCTG
ACCAAATCCCTGGTGGCGACCTCTCAAGTGGAAGACCTGGTGGTGAACCTGGTGCCGCTGGGC
CGCTAA

PP7-YG-PP7:
ATGAGCAAAACCATTGTTCTGAGCGTGGGTGAAGCGACCCGTACCCTGACCGAAATCCAGAGCA
CCGCTGACCGTCAAATTTTTGAGGAAAAAGTGGGTCCGCTGGTTGGCCGTCTGCGTCTGACCGC
GAGCCTGCGTCAGAACGGTGCGAAGACCGCGTACCGTGTGAACCTGAAACTGGACCAAGCGGA
TGTGGTTGATTGCAGCACCAGCGTTTGCGGCGAGCTGCCGAAAGTGCGTTACACCCAGGTTTGG
AGCCACGATGTGACCATCGTTGCGAACAGCACCGAAGCGAGCCGTAAGAGCCTGTATGATCTGA
CCAAAAGCCTGGTGGCGACCAGCCAAGTTGAGGACCTGGTGGTTAATCTGGTACCACTTGGTCG
C**TATGGC**TCGAAAACAATTGTCCTGTCCGTGGGCGAAGCAACCCGCACCCTGACCGAAATCCAA
TCTACCGCAGACCGCCAAATCTTTGAAGAAAAAGTGGGTCCGCTGGTCGGTCGTCTGCGTCTGA
CCGCCTCTCTGCGTCAGAACGGCGCGAAAACGGCCTATCGCGTCAATCTGAAACTGGATCAAGC

AGACGTGGTTGATTGCAGCACCTCTGTTTGTGGTGAACTGCCGAAAGTGCGTTATACGCAGGTTT
GGTCACATGACGTCACCATTGTGGCAAACTCGACGGAAGCTAGTCGCAAATCCCTGTACGATCTG
ACCAAATCCCTGGTGGCGACCTCTCAAGTGGAAGACCTGGTGGTGAACCTGGTGCCGCTGGGC
CGCTAA

PP7-GG-PP7:

ATGAGCAAAACCATTGTTCTGAGCGTGGGTGAAGCGACCCGTACCCTGACCGAAATCCAGAGCA
CCGCTGACCGTCAAATTTTTGAGGAAAAAGTGGGTCCGCTGGTTGGCCGTCTGCGTCTGACCGC
GAGCCTGCGTCAGAACGGTGCGAAGACCGCGTACCGTGTGAACCTGAAACTGGACCAAGCGGA
TGTGGTTGATTGCAGCACCAGCGTTTGCGGCGAGCTGCCGAAAGTGCGTTACACCCAGGTTTGG
AGCCACGATGTGACCATCGTTGCGAACAGCACCGAAGCGAGCCGTAAGAGCCTGTATGATCTGA
CCAAAAGCCTGGTGGCGACCAGCCAAGTTGAGGACCTGGTGGTTAATCTGGTACCACTTGGTCG
C**GGCGGT**TCGAAAACAATTGTCCTGTCCGTGGGCGAAGCAACCCGCACCCTGACCGAAATCCAA
TCTACCGCAGACCGCCAAATCTTTGAAGAAAAAGTGGGTCCGCTGGTCGGTCGTCTGCGTCTGA
CCGCCTCTCTGCGTCAGAACGGCGCGAAAACGGCCTATCGCGTCAATCTGAAACTGGATCAAGC
AGACGTGGTTGATTGCAGCACCTCTGTTTGTGGTGAACTGCCGAAAGTGCGTTATACGCAGGTTT
GGTCACATGACGTCACCATTGTGGCAAACTCGACGGAAGCTAGTCGCAAATCCCTGTACGATCTG
ACCAAATCCCTGGTGGCGACCTCTCAAGTGGAAGACCTGGTGGTGAACCTGGTGCCGCTGGGC
CGCTAA

PP7-AYG-PP7:

ATGAGCAAAACCATTGTTCTGAGCGTGGGTGAAGCGACCCGTACCCTGACCGAAATCCAGAGCA
CCGCTGACCGTCAAATTTTTGAGGAAAAAGTGGGTCCGCTGGTTGGCCGTCTGCGTCTGACCGC
GAGCCTGCGTCAGAACGGTGCGAAGACCGCGTACCGTGTGAACCTGAAACTGGACCAAGCGGA
TGTGGTTGATTGCAGCACCAGCGTTTGCGGCGAGCTGCCGAAAGTGCGTTACACCCAGGTTTGG
AGCCACGATGTGACCATCGTTGCGAACAGCACCGAAGCGAGCCGTAAGAGCCTGTATGATCTGA
CCAAAAGCCTGGTGGCGACCAGCCAAGTTGAGGACCTGGTGGTTAATCTGGTACCACTTGGTCG
C**GCATATGGC**TCGAAAACAATTGTCCTGTCCGTGGGCGAAGCAACCCGCACCCTGACCGAAATC
CAATCTACCGCAGACCGCCAAATCTTTGAAGAAAAAGTGGGTCCGCTGGTCGGTCGTCTGCGTC
TGACCGCCTCTCTGCGTCAGAACGGCGCGAAAACGGCCTATCGCGTCAATCTGAAACTGGATCA
AGCAGACGTGGTTGATTGCAGCACCTCTGTTTGTGGTGAACTGCCGAAAGTGCGTTATACGCAG
GTTTGGTCACATGACGTCACCATTGTGGCAAACTCGACGGAAGCTAGTCGCAAATCCCTGTACGA
TCTGACCAAATCCCTGGTGGCGACCTCTCAAGTGGAAGACCTGGTGGTGAACCTGGTGCCGCTG
GGCCGCTAA

PP7-YGG-PP7:

ATGAGCAAAACCATTGTTCTGAGCGTGGGTGAAGCGACCCGTACCCTGACCGAAATCCAGAGCA
CCGCTGACCGTCAAATTTTTGAGGAAAAAGTGGGTCCGCTGGTTGGCCGTCTGCGTCTGACCGC
GAGCCTGCGTCAGAACGGTGCGAAGACCGCGTACCGTGTGAACCTGAAACTGGACCAAGCGGA
TGTGGTTGATTGCAGCACCAGCGTTTGCGGCGAGCTGCCGAAAGTGCGTTACACCCAGGTTTGG
AGCCACGATGTGACCATCGTTGCGAACAGCACCGAAGCGAGCCGTAAGAGCCTGTATGATCTGA
CCAAAAGCCTGGTGGCGACCAGCCAAGTTGAGGACCTGGTGGTTAATCTGGTACCACTTGGTCG
C**TATGGCGGT**TCGAAAACAATTGTCCTGTCCGTGGGCGAAGCAACCCGCACCCTGACCGAAATC
CAATCTACCGCAGACCGCCAAATCTTTGAAGAAAAAGTGGGTCCGCTGGTCGGTCGTCTGCGTC
TGACCGCCTCTCTGCGTCAGAACGGCGCGAAAACGGCCTATCGCGTCAATCTGAAACTGGATCA
AGCAGACGTGGTTGATTGCAGCACCTCTGTTTGTGGTGAACTGCCGAAAGTGCGTTATACGCAG
GTTTGGTCACATGACGTCACCATTGTGGCAAACTCGACGGAAGCTAGTCGCAAATCCCTGTACGA
TCTGACCAAATCCCTGGTGGCGACCTCTCAAGTGGAAGACCTGGTGGTGAACCTGGTGCCGCTG
GGCCGCTAA

PP7-AA-PP7:

ATGAGCAAAACCATTGTTCTGAGCGTGGGTGAAGCGACCCGTACCCTGACCGAAATCCAGAGCA
CCGCTGACCGTCAAATTTTTGAGGAAAAAGTGGGTCCGCTGGTTGGCCGTCTGCGTCTGACCGC
GAGCCTGCGTCAGAACGGTGCGAAGACCGCGTACCGTGTGAACCTGAAACTGGACCAAGCGGA
TGTGGTTGATTGCAGCACCAGCGTTTGCGGCGAGCTGCCGAAAGTGCGTTACACCCAGGTTTGG
AGCCACGATGTGACCATCGTTGCGAACAGCACCGAAGCGAGCCGTAAGAGCCTGTATGATCTGA
CCAAAAGCCTGGTGGCGACCAGCCAAGTTGAGGACCTGGTGGTTAATCTTGTACCACTTGGTCG
C**GCAGCG**TCGAAAACCATCGTCCTGTCCGTGGGCGAAGCAACCCGCACCCTGACCGAAATCCAA

TCTACCGCAGACCGCCAAATCTTTGAAGAAAAAGTGGGTCCGCTGGTCGGTCGTCTGCGTCTGA
CCGCCTCTCTGCGTCAGAACGGCGCGAAAACGGCCTATCGCGTCAATCTGAAACTGGATCAAGC
AGACGTGGTTGATTGCAGCACCTCTGTTTGTGGTGAACTGCCGAAAGTGCGTTATACGCAGGTTT
GGTCACATGACGTCACCATTGTGGCAAACTCGACGGAAGCTAGTCGCAAATCCCTGTACGATCTG
ACCAAATCCCTGGTGGCGACCTCTCAAGTGGAAGACCTGGTGGTGAACCTGGTGCCGCTGGGC
CGCTAA

PP7-AAA-PP7:
ATGAGCAAAACCATTGTTCTGAGCGTGGGTGAAGCGACCCGTACCCTGACCGAAATCCAGAGCA
CCGCTGACCGTCAAATTTTTGAGGAAAAAGTGGGTCCGCTGGTTGGCCGTCTGCGTCTGACCGC
GAGCCTGCGTCAGAACGGTGCGAAGACCGCGTACCGTGTGAACCTGAAACTGGACCAAGCGGA
TGTGGTTGATTGCAGCACCAGCGTTTGCGGCGAGCTGCCGAAAGTGCGTTACACCCAGGTTTGG
AGCCACGATGTGACCATCGTTGCGAACAGCACCGAAGCGAGCCGTAAGAGCCTGTATGATCTGA
CCAAAAGCCTGGTGGCGACCAGCCAAGTTGAGGACCTGGTGGTTAATCTTGTACCACTTGGTCG
C**GCAGCTGCG**TCGAAAACCATCGTCCTGTCCGTGGGCGAAGCAACCCGCACCCTGACCGAAATC
CAATCTACCGCAGACCGCCAAATCTTTGAAGAAAAAGTGGGTCCGCTGGTCGGTCGTCTGCGTC
TGACCGCCTCTCTGCGTCAGAACGGCGCGAAAACGGCCTATCGCGTCAATCTGAAACTGGATCA
AGCAGACGTGGTTGATTGCAGCACCTCTGTTTGTGGTGAACTGCCGAAAGTGCGTTATACGCAG
GTTTGGTCACATGACGTCACCATTGTGGCAAACTCGACGGAAGCTAGTCGCAAATCCCTGTACGA
TCTGACCAAATCCCTGGTGGCGACCTCTCAAGTGGAAGACCTGGTGGTGAACCTGGTGCCGCTG
GGCCGCTAA

PP7-AAAA-PP7:
ATGAGCAAAACCATTGTTCTGAGCGTGGGTGAAGCGACCCGTACCCTGACCGAAATCCAGAGCA
CCGCTGACCGTCAAATTTTTGAGGAAAAAGTGGGTCCGCTGGTTGGCCGTCTGCGTCTGACCGC
GAGCCTGCGTCAGAACGGTGCGAAGACCGCGTACCGTGTGAACCTGAAACTGGACCAAGCGGA
TGTGGTTGATTGCAGCACCAGCGTTTGCGGCGAGCTGCCGAAAGTGCGTTACACCCAGGTTTGG
AGCCACGATGTGACCATCGTTGCGAACAGCACCGAAGCGAGCCGTAAGAGCCTGTATGATCTGA
CCAAAAGCCTGGTGGCGACCAGCCAAGTTGAGGACCTGGTGGTTAATCTTGTACCACTTGGTCG
C**GCAGCAGCTGCG**TCGAAAACCATCGTCCTGTCCGTGGGCGAAGCAACCCGCACCCTGACCGA
AATCCAATCTACCGCAGACCGCCAAATCTTTGAAGAAAAAGTGGGTCCGCTGGTCGGTCGTCTGC
GTCTGACCGCCTCTCTGCGTCAGAACGGCGCGAAAACGGCCTATCGCGTCAATCTGAAACTGGA
TCAAGCAGACGTGGTTGATTGCAGCACCTCTGTTTGTGGTGAACTGCCGAAAGTGCGTTATACGC
AGGTTTGGTCACATGACGTCACCATTGTGGCAAACTCGACGGAAGCTAGTCGCAAATCCCTGTAC
GATCTGACCAAATCCCTGGTGGCGACCTCTCAAGTGGAAGACCTGGTGGTGAACCTGGTGCCGC
TGGGCCGCTAA

PP7-AAAAA-PP7:
ATGAGCAAAACCATTGTTCTGAGCGTGGGTGAAGCGACCCGTACCCTGACCGAAATCCAGAGCA
CCGCTGACCGTCAAATTTTTGAGGAAAAAGTGGGTCCGCTGGTTGGCCGTCTGCGTCTGACCGC
GAGCCTGCGTCAGAACGGTGCGAAGACCGCGTACCGTGTGAACCTGAAACTGGACCAAGCGGA
TGTGGTTGATTGCAGCACCAGCGTTTGCGGCGAGCTGCCGAAAGTGCGTTACACCCAGGTTTGG
AGCCACGATGTGACCATCGTTGCGAACAGCACCGAAGCGAGCCGTAAGAGCCTGTATGATCTGA
CCAAAAGCCTGGTGGCGACCAGCCAAGTTGAGGACCTGGTGGTTAATCTTGTACCACTTGGTCG
C**GCAGCCGCAGCTGCG**TCGAAAACCATCGTCCTGTCCGTGGGCGAAGCAACCCGCACCCTGAC
CGAAATCCAATCTACCGCAGACCGCCAAATCTTTGAAGAAAAAGTGGGTCCGCTGGTCGGTCGT
CTGCGTCTGACCGCCTCTCTGCGTCAGAACGGCGCGAAAACGGCCTATCGCGTCAATCTGAAAC
TGGATCAAGCAGACGTGGTTGATTGCAGCACCTCTGTTTGTGGTGAACTGCCGAAAGTGCGTTAT
ACGCAGGTTTGGTCACATGACGTCACCATTGTGGCAAACTCGACGGAAGCTAGTCGCAAATCCCT
GTACGATCTGACCAAATCCCTGGTGGCGACCTCTCAAGTGGAAGACCTGGTGGTGAACCTGGTG
CCGCTGGGCCGCTAA

PP7-GS-PP7:
ATGAGCAAAACCATTGTTCTGAGCGTGGGTGAAGCGACCCGTACCCTGACCGAAATCCAGAGCA
CCGCTGACCGTCAAATTTTTGAGGAAAAAGTGGGTCCGCTGGTTGGCCGTCTGCGTCTGACCGC
GAGCCTGCGTCAGAACGGTGCGAAGACCGCGTACCGTGTGAACCTGAAACTGGACCAAGCGGA
TGTGGTTGATTGCAGCACCAGCGTTTGCGGCGAGCTGCCGAAAGTGCGTTACACCCAGGTTTGG
AGCCACGATGTGACCATCGTTGCGAACAGCACCGAAGCGAGCCGTAAGAGCCTGTATGATCTGA

CCAAAAGCCTGGTGGCGACCAGCCAAGTTGAGGACCTGGTGGTTAATCTTGTACCACTTGGTCG
C**GGTAGT**TCGAAAACCATCGTCCTGTCCGTGGGCGAAGCAACCCGCACCCTGACCGAAATCCAA
TCTACCGCAGACCGCCAAATCTTTGAAGAAAAAGTGGGTCCGCTGGTCGGTCGTCTGCGTCTGA
CCGCCTCTCTGCGTCAGAACGGCGCGAAAACGGCCTATCGCGTCAATCTGAAACTGGATCAAGC
AGACGTGGTTGATTGCAGCACCTCTGTTTGTGGTGAACTGCCGAAAGTGCGTTATACGCAGGTTT
GGTCACATGACGTCACCATTGTGGCAAACTCGACGGAAGCTAGTCGCAAATCCCTGTACGATCTG
ACCAAATCCCTGGTGGCGACCTCTCAAGTGGAAGACCTGGTGGTGAACCTGGTGCCGCTGGGC
CGCTAA

PP7-GSG-PP7:
ATGAGCAAAACCATTGTTCTGAGCGTGGGTGAAGCGACCCGTACCCTGACCGAAATCCAGAGCA
CCGCTGACCGTCAAATTTTTGAGGAAAAAGTGGGTCCGCTGGTTGGCCGTCTGCGTCTGACCGC
GAGCCTGCGTCAGAACGGTGCGAAGACCGCGTACCGTGTGAACCTGAAACTGGACCAAGCGGA
TGTGGTTGATTGCAGCACCAGCGTTTGCGGCGAGCTGCCGAAAGTGCGTTACACCCAGGTTTGG
AGCCACGATGTGACCATCGTTGCGAACAGCACCGAAGCGAGCCGTAAGAGCCTGTATGATCTGA
CCAAAAGCCTGGTGGCGACCAGCCAAGTTGAGGACCTGGTGGTTAATCTTGTACCACTTGGTCG
C**GGTAGTGGA**TCGAAAACCATCGTCCTGTCCGTGGGCGAAGCAACCCGCACCCTGACCGAAATC
CAATCTACCGCAGACCGCCAAATCTTTGAAGAAAAAGTGGGTCCGCTGGTCGGTCGTCTGCGTC
TGACCGCCTCTCTGCGTCAGAACGGCGCGAAAACGGCCTATCGCGTCAATCTGAAACTGGATCA
AGCAGACGTGGTTGATTGCAGCACCTCTGTTTGTGGTGAACTGCCGAAAGTGCGTTATACGCAG
GTTTGGTCACATGACGTCACCATTGTGGCAAACTCGACGGAAGCTAGTCGCAAATCCCTGTACGA
TCTGACCAAATCCCTGGTGGCGACCTCTCAAGTGGAAGACCTGGTGGTGAACCTGGTGCCGCTG
GGCCGCTAA

PP7-GSGS-PP7:
ATGAGCAAAACCATTGTTCTGAGCGTGGGTGAAGCGACCCGTACCCTGACCGAAATCCAGAGCA
CCGCTGACCGTCAAATTTTTGAGGAAAAAGTGGGTCCGCTGGTTGGCCGTCTGCGTCTGACCGC
GAGCCTGCGTCAGAACGGTGCGAAGACCGCGTACCGTGTGAACCTGAAACTGGACCAAGCGGA
TGTGGTTGATTGCAGCACCAGCGTTTGCGGCGAGCTGCCGAAAGTGCGTTACACCCAGGTTTGG
AGCCACGATGTGACCATCGTTGCGAACAGCACCGAAGCGAGCCGTAAGAGCCTGTATGATCTGA
CCAAAAGCCTGGTGGCGACCAGCCAAGTTGAGGACCTGGTGGTTAATCTTGTACCACTTGGTCG
C**GGTAGTGGATCA**TCGAAAACCATCGTCCTGTCCGTGGGCGAAGCAACCCGCACCCTGACCGAA
ATCCAATCTACCGCAGACCGCCAAATCTTTGAAGAAAAAGTGGGTCCGCTGGTCGGTCGTCTGC
GTCTGACCGCCTCTCTGCGTCAGAACGGCGCGAAAACGGCCTATCGCGTCAATCTGAAACTGGA
TCAAGCAGACGTGGTTGATTGCAGCACCTCTGTTTGTGGTGAACTGCCGAAAGTGCGTTATACGC
AGGTTTGGTCACATGACGTCACCATTGTGGCAAACTCGACGGAAGCTAGTCGCAAATCCCTGTAC
GATCTGACCAAATCCCTGGTGGCGACCTCTCAAGTGGAAGACCTGGTGGTGAACCTGGTGCCGC
TGGGCCGCTAA

3. Supplemental Figures

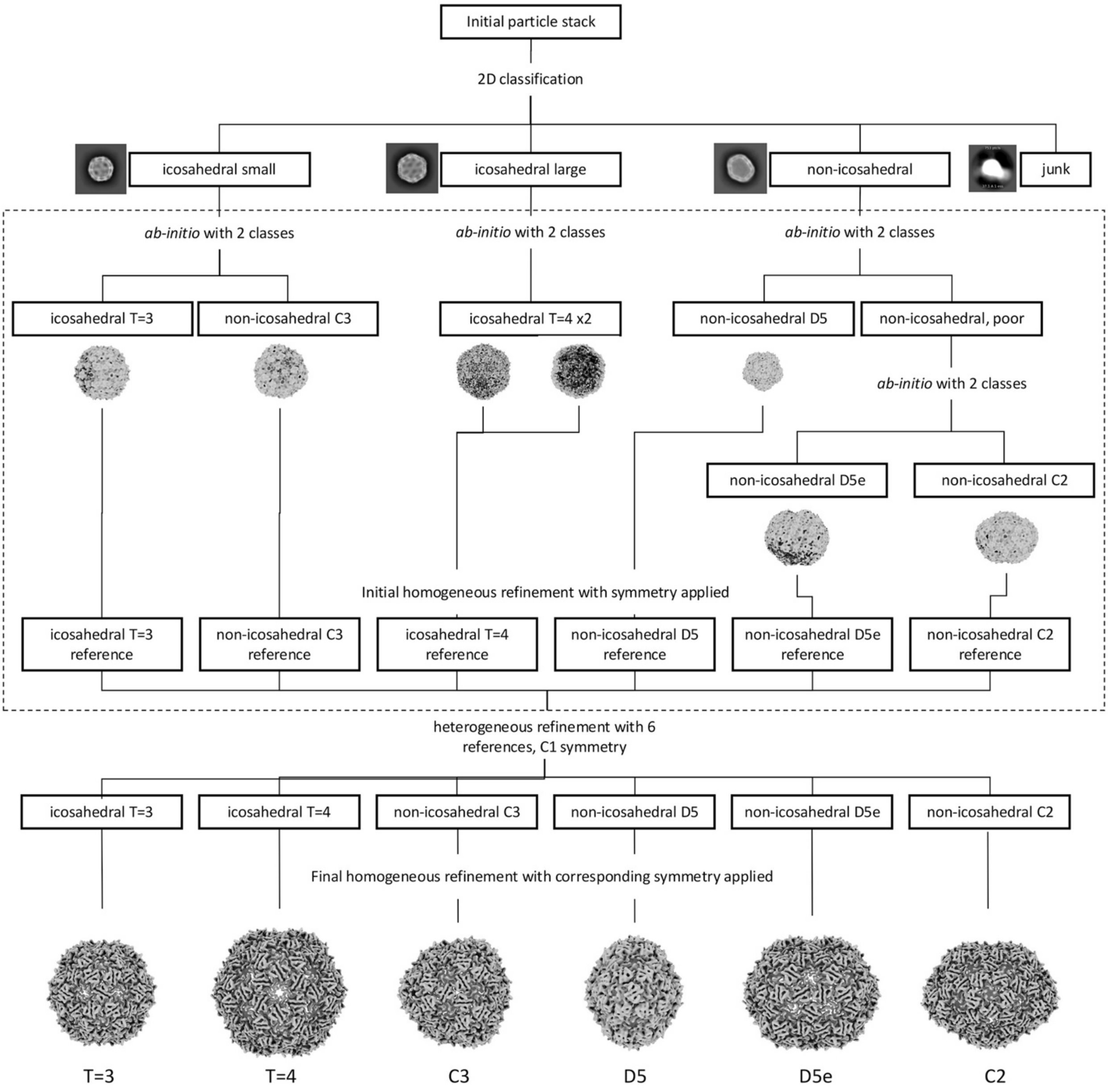

Figure S1. Cryo-EM processing workflow.

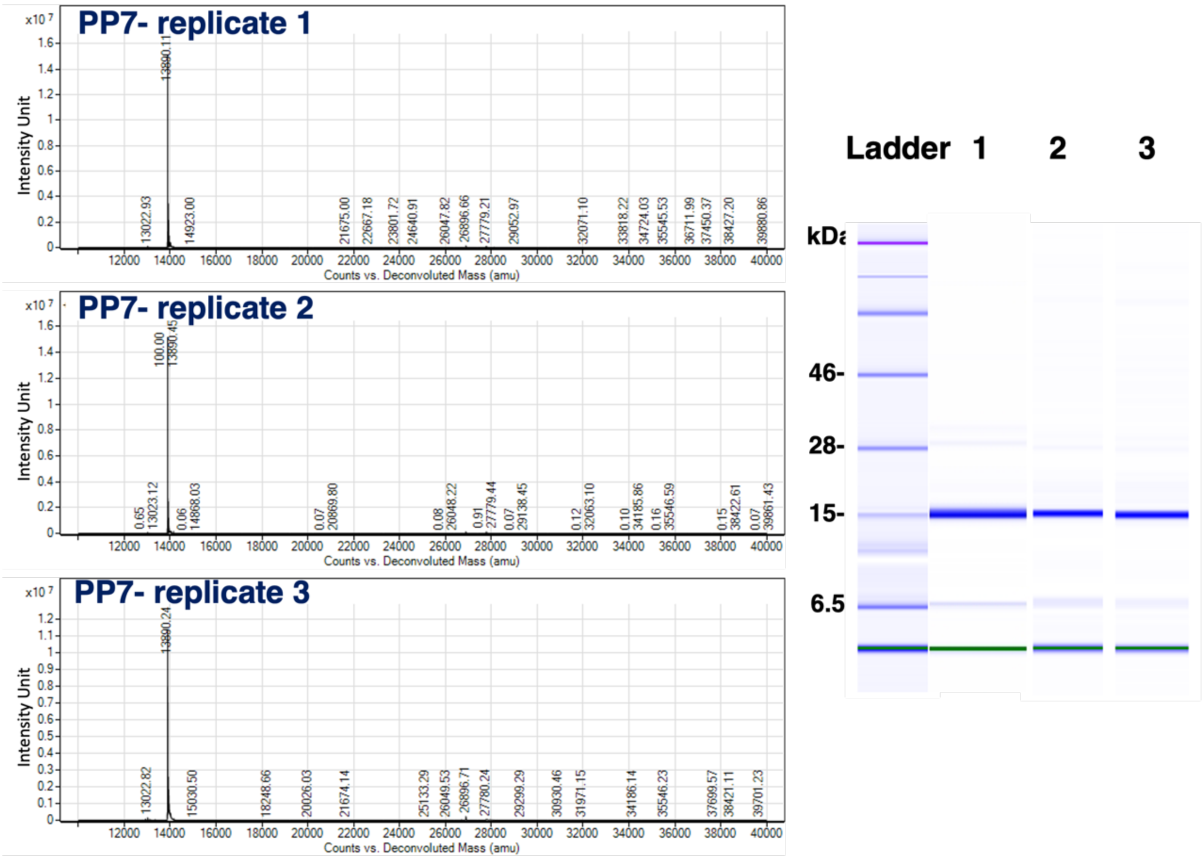

Figure S2. LC-MS (left) and microfluidics gel electrophoresis (right) characterization of three replicates of PP7 VLPs.

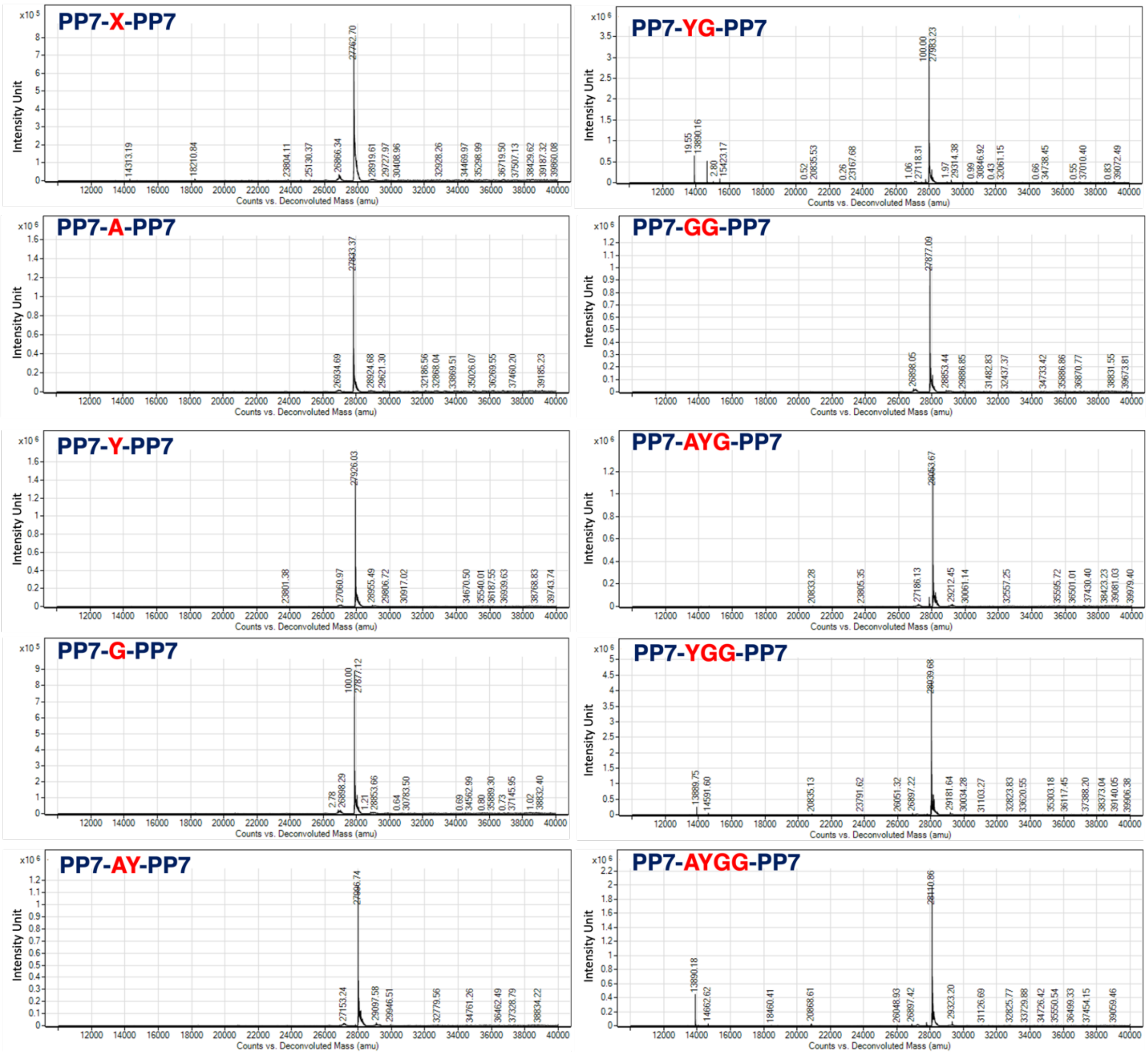

Figure S3. LC-MS characterization of PP7 dimer constructs consisting of 0-4 amino acid linkers.

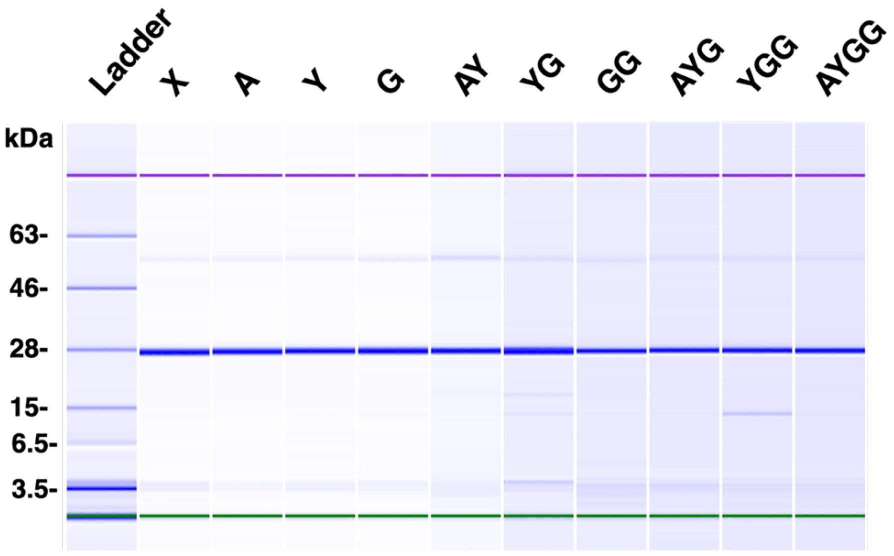

Figure S4. microfluidics gel electrophoresis characterization of PP7 dimer constructs consisting of 0-4 amino acid linkers.

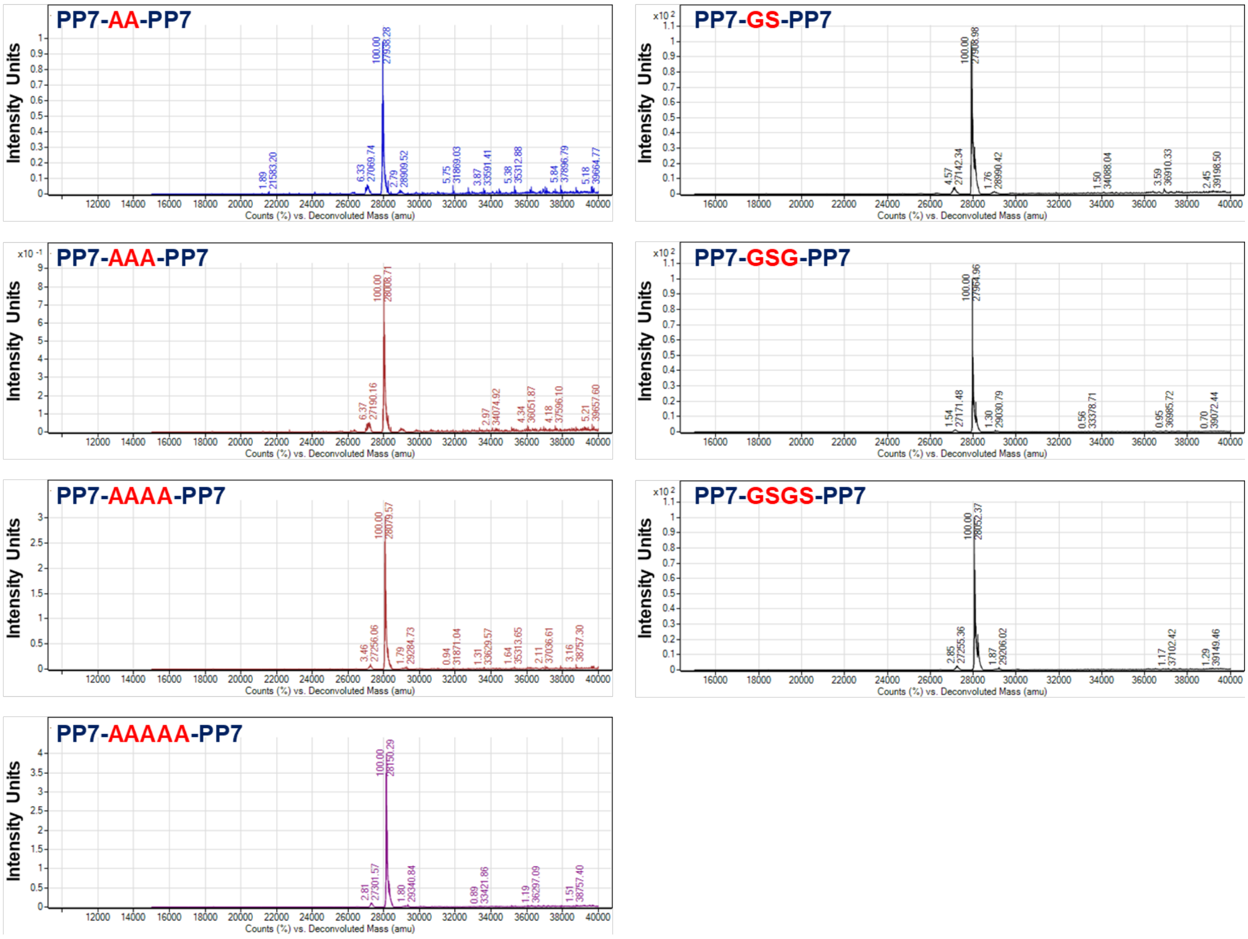

Figure S5. LC-MS characterization of additional PP7 dimer constructs with either poly-alanine or alternative glycine-serine amino acid linkers.

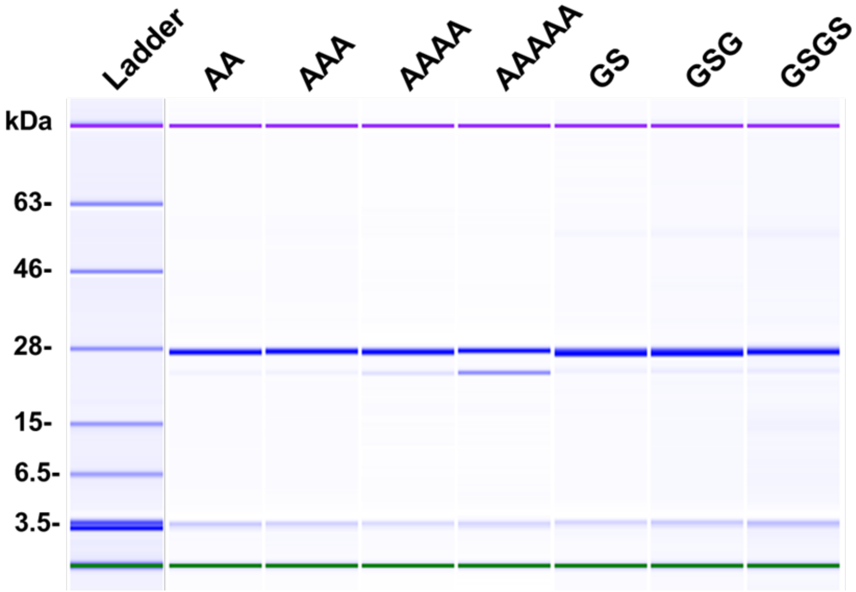

Figure S6. microfluidics gel electrophoresis characterization of additional PP7 dimer constructs with either poly-alanine or alternative glycine-serine amino acid linkers.

4. References

[1] L. Zhao, M. Kopylov, C.S. Potter, B. Carragher, M.G. Finn, *ACS Nano* **2019**, *13*, 4443-4454. https://doi.org/10.1021/acsnano.8b09683

[2] P. Keshavarz-Joud, L. Zhao, D. Bobe, C. Hernandez, M. Kopylov, L.Y. Yen, N. Djeddar, B. Thompson, C. Connors, G. Gibson, A. Bryksin, M.G. Finn, *ACS Nano* **2023**, *17*, 18470-18480. https://doi.org/10.1021/acsnano.3c06178

[3] D. Tegunov, P. Cramer, *Nature methods* **2019**, *16*, 1146-1152.

[4] S. Zheng, G. Wolff, G. Greenan, Z. Chen, F.G.A. Faas, M. Bárcena, A.J. Koster, Y. Cheng, D.A. Agard, *J Struct Biol X* **2022**, *6*, 100068. https://doi.org/10.1016/j.yjsbx.2022.100068

[5] A. Martinez-Sanchez, I. Garcia, S. Asano, V. Lucic, J.J. Fernandez, *J Struct Biol* **2014**, *186*, 49-61. https://doi.org/10.1016/j.jsb.2014.02.015

[6] J.R. Kremer, D.N. Mastronarde, J.R. McIntosh, *J Struct Biol* **1996**, *116*, 71-76. https://doi.org/10.1006/jsbi.1996.0013
